# Adsorption of diclofenac onto different biochar microparticles: Dataset – Characterization and dosage of biochar

**DOI:** 10.1016/j.dib.2017.10.041

**Published:** 2017-10-21

**Authors:** Linson Lonappan, Tarek Rouissi, Satinder Kaur Brar, Mausam Verma, Rao Y. Surampalli

**Affiliations:** a INRS-ETE, Université du Québec, 490, Rue de la Couronne, Québec, Canada G1K 9A9; b CO2 Solutions Inc., 2300, Rue Jean-Perrin, Québec, Canada G2C 1T9; c Department of Civil Engineering, University of Nebraska-Lincoln, N104 SEC P.O. Box 886105, Lincoln, NE 68588-6105, United States

**Keywords:** Adsorption, Diclofenac, Biochar, Characterization

## Abstract

Due to its wide occurrence in water resources and toxicity, pharmaceuticals and personal care products are becoming an emerging concern throughout the world. Application of residual/waste materials for water remediation can be a good strategy in waste management as well as in waste valorization. Herein, this dataset provides information on biochar application for the removal of emerging contaminant, diclofenac from water matrices. The data presented here is an extension of the research article explaining the mechanisms of adsorption diclofenac on biochars (Lonappan et al., 2017 [1]). This data article provides general information on the surface features of pine wood and pig manure biochar with the help of SEM and FTIR data. This dataset also provides information on XRD profiles of pine wood and pig manure biochars. In addition, different amounts of biochars were used to study the removal of a fixed concentration of diclofenac and the data is provided with this data set.

**Specifications Table**

**Table:** 

Subject area	Chemistry/Chemical engineering
More specific subject area	Adsorption, Surface Chemistry, Environmental Engineering
Type of data	Table, image (XRD, SEM)), text file, figure(FTIR)
How data was acquired	SEM: Zeiss Evo®50 Smart SEM
	FTIR: Perkin Elmer, Spectrum RXI, FT-IR instrument fitted with lithium tantalate (LiTaO_3_) detector
	XRD: Panalytical Empyrean XRD with monochromatized CuK alfa radiation (1.5418A).
	LDTD-MS/MS: Concentrations of diclofenac was measured using LDTD-APCI (atmospheric pressure chemical ionization) source (LDTD T-960, Phytronix Technologies, Quebec, Canada) mounted on a TSQ Quantum access triple quadruple mass spectrometer (Thermo Scientific, Mississauga, Ontario, Canada)
Data format	Pre-processed and analyzed
Experimental factors	Biochar samples (from pinewood and pig manure) were grounded to obtain microparticles and the data here is given is for characterization of biochar. Moreover, data for dosage effect of biochar on adsorption for diclofenac is given.
Experimental features	Characterization data of biochar microparticles obtained from SEM, XRD, and FTIR are given.
	Adsorption studies were carried out for the removal of diclofenac using biochar microparticles. Various biochar dosages ranging from 1 g L^−1^ to 20 g L^−1^were tested.
Data source location	Bioprocessing and NanoEnzyme Formulation Facility (BANEFF), INRS-ETE, Université du Québec, 490, Rue de la Couronne, Québec, Canada G1K 9A9
Data accessibility	Data presented in this article
Related research article	The associated research article related to this data set is [1]

**Value of the data**

• Characterization data for biochar derived from two different feedstock (pine wood and pig manure) are given.
• Dataset provides an insight to the surface features of biochar.
• Dataset gives information on the adsorption capacity of biochar for emerging contaminant diclofenac.
• Dataset would be useful to identify the dosage effect of biochar on the adsorption of diclofenac.

## 1 Data

**Fig. 1:**
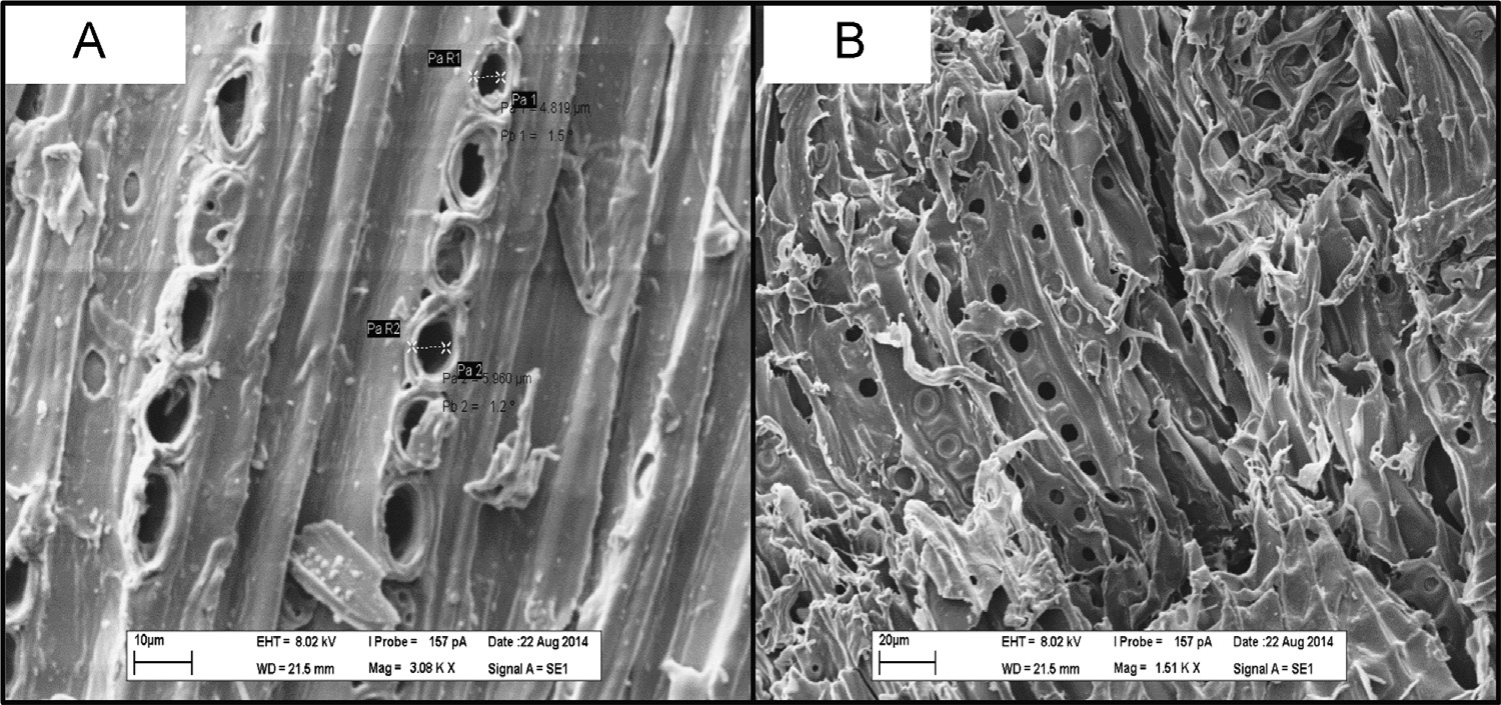
Scanning electron micrographs of biochar: (A) Pinewood biochar (BC-PW), (B) Pig Manure biochar (BC-PM).

**Fig. 2:**
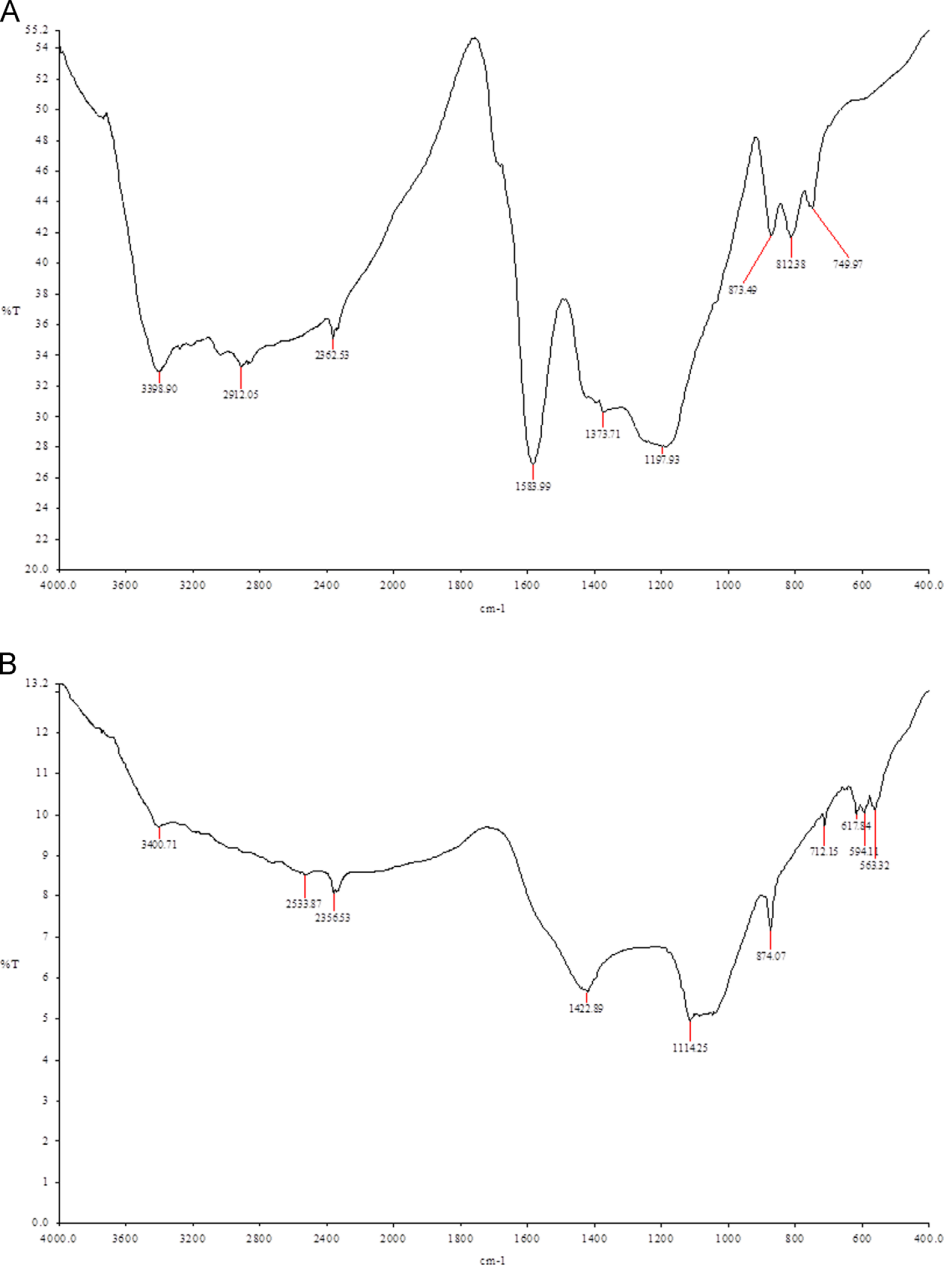
Fourier transform infra-red spectra and of biochar; BC-PW: pine wood biochar, BC-PM: pig manure biochar.

**Fig. 3:**
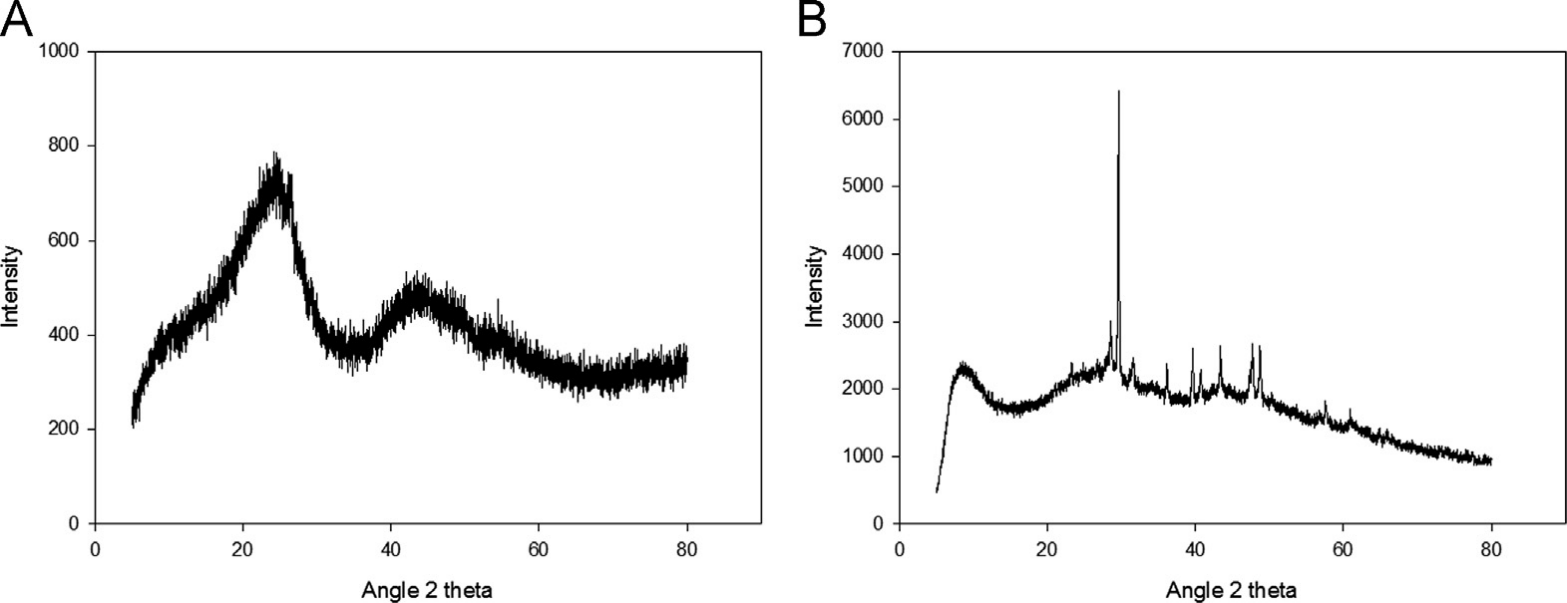
X-ray diffraction curve of biochar; BC-PW: pine wood biochar, BC-PM: pig manure biochar.

The dataset comprises characterization as well as experimental data. Fig. 1 presents the scanning electron micrographs (SEM) of pine wood and pig manure biochar microparticles. Fig. 2. presents Fourier-transform infrared spectroscopy (FTIR) images of biochar microparticles. Fig. 3 presents X-ray Diffraction (XRD) images of biochar microparticles. Table 1shows the effect of adsorbent dosage on the removal of diclofenac and removal efficiency.

**Table 1:** Effect of adsorbent dosage on adsorption amount and removal efficiency.

**Adsorbent dosage (g L**^−^^**1**^**)**	**Removal efficiency (%)**		**Adsorption amount on biochar (µg g**^−^^**1**^**)**	
	**BC-PW**	**BC-PM**	**BC-PW**	**BC-PM**
2	42.99	95.87	107.49	239.69
6	83.16	100	69.30	–
10	93.67	100	46.83	–
14	96.52	100	34.47	–
20	98.81	100	24.70	–

^*^BC-PW: Pinewood biochar microparticles, BC-PM: Pig manure biochar microparticles.

## 2 Experimental design, materials and methods

### 2.1 Biochar microparticle preparation

Two types of biochars were prepared from pinewood and pig manure and named as BC-PW and BC-PM, respectively. Preparation of biochar and microparticles are explained elsewhere [1], [2].

### 2.2 Characterization of biochar microparticles

Scanning electron micrographs of the biochar microparticles are recorded using Zeiss Evo®50 Smart SEM system. FTIR spectra of the adsorbents were recorded using Perkin Elmer, Spectrum RXI, FT-IR instrument fitted with lithium tantalate (LiTaO_3_) detector. XRD spectra of the adsorbents were recorded using Panalytical Empyrean XRD fitted with monochromatized CuK alfa radiation (1.5418A).

### 2.3 Adsorption studies

Adsorption studies were carried out using 50 mg (1 g L^−1^), 0.1 g (2 g L^−^^1^), 0.3 g (6 g L^−^^1^), 0.5 g (10 g L^−1^), 0.7 g (14 g L^−^^1^) and 1 g (20 g L^−^^1^) of biochar samples with 50 mL of 500 µg L^−1^ of diclofenac (DCF). Batch adsorption studies were carried out in an INFORS HT – multitron standard shaking incubator (INFORS, Mississauga, Canada). Experimental conditions are as follows – shaking speed: 200 rpm; temperature: 25 ± 1 °C, pH: 6.5, centrifugation (after adsorption studies): at 11,600×*g* for 10 min in a MiniSpin^®^ plus centrifuge. The supernatant was analyzed for remaining DCF using LDTD-MS/MS [3].

It was observed that for both BC-PW and BC-PM, increasing the adsorbent dosage considerably enhanced the removal efficiency. BC-PM possessed better adsorbent properties than BC-PW and showed higher potential for the removal of DCF compared to BC-PW. With a dosage of 2 g L^−1^, BC-PM achieved a removal efficiency of 95.87% and above 2 g L^−1^ dosage level, BC-PM always achieved nearly 100% removal efficiency. For BC-PW, removal efficiency increased from 43% to 98.8% with a dosage varying from 2 to 20 g L^−1^. However, the adsorption amount (µg g^−^^1^) on biochar decreased with increase in adsorbent dosage. This observation can be explained as a consequence of partial aggregation of biochar at higher concentrations of biochar which will decrease the active sites on the surface of biochar [4], [5]. Adsorbent dosage experiment was carried out at equilibrium time and samples were drawn. In the case of BC-PW, the complete removal might have been obtained during any time of the adsorption. Therefore, adsorption amount cannot be considered as the equilibrium adsorption capacity of the biochar BC-PM. As shown in Fig. 1, porous structure of biochars probably had a positive effect on the adsorption of DCF [6]. Moreover, as shown in Fig. 2, both biochars are rich in surface functional groups which in turn can facilitate the adsorption.
